# The Mediating Roles of Self‐Disclosure and Social Support in the Relationship Between Self‐Perceived Burden and Benefit Finding Among Postoperative Colorectal Cancer Patients

**DOI:** 10.1002/nop2.70672

**Published:** 2026-07-01

**Authors:** Yi Long Zhou, Ya Jing Wang, Jing Jing Guo, Jia Wen, Hui Fang Guo

**Affiliations:** ^1^ Department of Nursing The First Affiliated Hospital of Dalian Medical University Dalian China; ^2^ Urology Department Zhongda Hospital, Southeast University Nanjing Jiangsu China; ^3^ Department of Nursing, Fudan University Shanghai Cancer Center; Department of Oncology, Shanghai Medical College Fudan University Shanghai China

**Keywords:** benefit finding, colorectal cancer, mediating effect, self‐disclosure, self‐perceived burden

## Abstract

**Aim:**

To explore the mediating roles of social support and self‐disclosure in the association between self‐perceived burden and benefit finding among postoperative colorectal cancer patients and to provide evidence that may inform nursing practice.

**Design:**

A cross‐sectional descriptive study.

**Methods:**

From November 2023 to June 2024, 165 patients who had undergone colorectal cancer surgery were recruited from a Class III Grade A hospital in China using convenience sampling. Data were collected using the Benefit Finding Scale, Self‐Perceived Burden Scale, Social Support Rating Scale, and The Distress Disclosure Index Scale. Correlation analysis and mediation effect tests were conducted.

**Results:**

Benefit finding was negatively correlated with self‐perceived burden (r = −0.687, *p* < 0.01) and positively correlated with self‐disclosure and social support (*r* = 0.712, 0.696, *p* < 0.01). Self‐perceived burden is associated with benefit finding through three mediating pathways: the mediating effect of social support was −0.193 (28.2% of the total effect); the mediating effect of self‐disclosure was −0.153 (22.3%); and the chain mediating effect of social support and self‐disclosure was −0.049 (7.1%).

**Patient or Public Contribution:**

No Patient or Public Contribution.

**Implications for Nursing Practice:**

This study suggests that assessment of self‐perceived burden may be considered as part of routine nursing assessment for postoperative colorectal cancer patients. Interventions aimed at enhancing social support and encouraging self‐disclosure may be worthy of further investigation, as these factors were associated with higher levels of benefit finding in this study.

## 1 Introduction

Colorectal cancer is a leading gastrointestinal malignancy worldwide, ranking third in cancer incidence and second in mortality. There are approximately 1.88 million new cases annually, accounting for 9.8% of all cancer incidences, and around 910,000 deaths, constituting 9.2% of all cancer mortalities (Sung et al. 2021). Currently, radical surgery is one of the most efficacious clinical treatments for colorectal cancer. By excising the cancerous part and clearing the regional lymph nodes, it can effectively control the proliferation and differentiation of tumour cells, reduce the risks of recurrence and metastasis, and improve the overall survival rate of patients (Jin et al. 2020; Wang, Ding, et al. 2022). However, postoperative colorectal cancer patients often encounter numerous problems such as an excessive self‐perceived burden and fear of disease progression (Hu et al. 2024; Zhang et al. 2022). With the development of positive psychology, the research perspective has shifted, and scholars have begun to explore the positive impacts of adversity on patients. Some scholars have discovered that when patients experience adversity, it can expedite their personal growth and potentially lead to positive changes to cope with the threats posed by the disease, a process known as benefit finding (BF) (Hamama‐Raz et al. 2021). Some studies have found that self‐perceived burden, as a significant stressor, is more likely to induce suicidal thoughts and behaviours than negative psychological factors like anxiety and depression and may be a potential influencing factor for benefit finding in colorectal cancer patients (Kanzler et al. 2012). Social support is a protective factor for mental health and has been proven to affect benefit finding in certain cancers (Jin et al. 2021). Self‐disclosure, as a successful self‐regulation method, can stimulate individuals to develop positive self‐perceptions and thus discover benefits from traumatic experiences (Li et al. 2020). Based on the stress and coping theory, when individuals face stressors such as cancer, they will make diverse cognitive evaluations and utilize existing coping resources or strategies to cope, thereby maintaining good physical and mental states (Taylor 1983). Against this theoretical framework, this study analysed the mediating effects of self‐disclosure and social support between self‐perceived burden and benefit finding, aiming to provide a reference for formulating intervention measures to enhance BF in postoperative colorectal cancer patients.

## 2 Theoretical Framework

**FIGURE 1:**
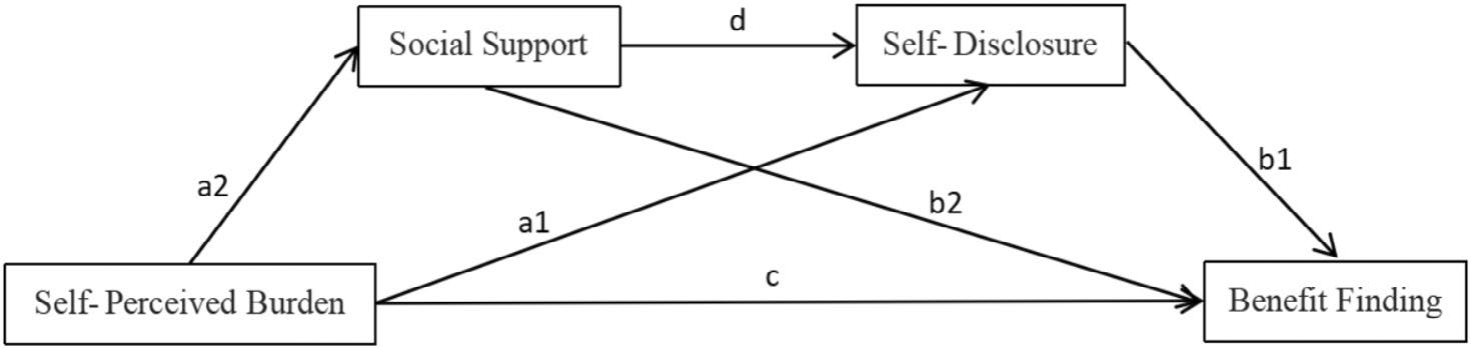
Hypothesized composite mediation model.

This study was guided by Lazarus and Folkman's Stress and Coping Theory (Taylor 1983). According to the theory, a stressor (colorectal cancer diagnosis) triggers cognitive appraisal and coping processes, which influence individuals adaptation outcomes. We operationalized self‐perceived burden (negative appraisal) and social support (positive appraisal) as cognitive appraisals, self‐disclosure as coping, and benefit finding as the outcome. A composite mediation model was hypothesized, including a direct effect, two independent indirect paths (via self‐disclosure alone and via social support alone), and a serial indirect path (self‐perceived burden → social support → self‐disclosure → benefit finding). The following hypotheses were tested (Figure 1).

- H1: Self‐perceived burden → benefit finding (direct negative effect, c)
- H2: Self‐perceived burden → self‐disclosure (negative, a1)
- H3: Self‐perceived burden → social support (negative, a2).
- H4: Self‐disclosure → benefit finding (positive, b1).
- H5: Social support → benefit finding (positive, b2).
- H6: Social support → self‐disclosure (positive, d).
- H7: Indirect effect via self‐disclosure only (a1*b1).
- H8: Indirect effect via social support only (a2*b2).
- H9: Serial indirect effect via social support → self‐disclosure (a2*d*b1).
- All hypotheses were directional; significance level ɑ = 0.05.

## 3 Materials and Methods

### 3.1 Participants

Participants were recruited via convenience sampling from inpatients who received colorectal cancer surgery at a tertiary Grade A hospital in China between November 2023 and June 2024. Inclusion criteria: ① Aged between 18 and 80 years old; ② Patients pathologically diagnosed with primary colorectal cancer and undergoing first‐time surgical treatment according to the “Chinese Colorectal Cancer Diagnosis and Treatment Guidelines (2023 Edition)” (Oncology Branch of Chinese Medical Association, and Department of Medical Administration, National Health Commission 2023); ③ Informed consent and voluntary participation in this study. Exclusion criteria: ① Critically ill patients unable to complete the survey; ② Patients with other malignant tumours; ③ Patients with a history of mental illness, cognitive impairment, and communication disorders. Prior to data collection, all potential participants received a comprehensive verbal and written explanation of the study purpose, procedures, potential benefits and risks, and the voluntary participation. Participants were explicitly informed that their decision to participate or decline would not affect their medical care or their relationship with healthcare providers, and that they could withdraw from the study at any time without giving a reason. All data were anonymized and processed confidentially to protect participant privacy. Copies of signed consent forms are archived by the corresponding author and can be made available upon request. Initially, 169 questionnaires were distributed. After excluding 4 invalid responses, 165 valid questionnaires were retained, yielding an effective response rate of 97.63%. This study was approved by the ethics committee of the First Affiliated Hospital of Dalian Medical University on January 30, 2024 (PJ‐KS‐KY‐2024‐74).

A sensitivity power analysis was conducted to assess whether the final sample size (*N* = 165) provided sufficient statistical power to detect the hypothesized indirect effects. The analysis employed a Monte Carlo simulation approach using the R package Web Power (version 0.9.4), with 5000 replications (Zhang and Yuan 2018). The following parameters were specified: α = 0.05 (two‐tailed), target power = 0.80, and the empirical standardized indirect effects derived from the observed data (via Self‐Disclosure: ab = −0.153; via Social Support: ab = −0.193). The results indicated that, given *N* = 165, the minimum detectable indirect effect size (absolute value) at 80% power was 0.09, which corresponds to a medium effect according to Cohen's (Cohen 1988) benchmarks. Both observed indirect effects (0.153 and 0.193) exceeded this threshold. Accordingly, the estimated statistical power for the indirect effect via Self‐Disclosure was 0.92, and that for the indirect effect via Social Support was 0.96. These results confirmed that the sample size of 165 was adequate for testing the mediation hypotheses.

### 3.2 Research Tools

#### 3.2.1 General Information Questionnaire

A self‐designed questionnaire includes basic information of patients, such as gender, age, educational level, monthly family income, marital status, payment method, cancer stage, disease classification, and presence or absence of stoma.

#### 3.2.2 Benefit Finding Scale (BFS)

This scale was adapted by Weaver et al. (Weaver et al. 2008), and translated into Chinese by Chinese scholar Liu Zhunzhun et al. (Liu et al. 2015). The scale consists of 6 dimensions, namely acceptance, family relationship, world view, personal growth, social relationship, and health behaviour, with a total of 22 items. A Likert 5‐point scale is used, ranging from “not at all” to “very much”, scored as 1–5 points respectively, and the total score ranges from 22 to 110 points. A higher total score indicates a higher level of BF in patients. In this study, the Cronbach's α coefficient was 0.88, indicating good reliability. The KMO value was 0.795, suggesting that the data were suitable for factor analysis.

#### 3.2.3 Self‐Perceived Burden Scale (SPBS)

This scale was developed by Cousineau et al. (Cousineau et al. 2003), in 2003 and translated and simplified into Chinese by Wu Yanyan et al. (Wu and Jiang 2010). Into 10 items, used to measure the burden of patients in three dimensions: economic, emotional, and physical. A Likert 5‐point scoring method is adopted (1 = never considered, 5 = always considered), and a higher score indicates a heavier SPB of the patient. A score < 20 indicates no obvious SPB, 20 ≤ score < 30 indicates mild SPB, 30 ≤ score < 40 indicates moderate SPB, and a score ≥ 40 indicates severe SPB. In this study, the Cronbach's α coefficient was 0.825, indicating good reliability. The KMO value was 0.883, confirming that the data were suitable for factor analysis.

#### 3.2.4 Social Support Rating Scale (SSRS)

The Social Support Rating Scale developed by Xiao Shuiyuan et al. (Dai 2010), was used, including three dimensions: objective support, subjective support, and utilization degree of support, with a total of 10 items. Scoring method of the scale: items 1–4 and 8–10 are single‐choice questions, item 5 is scored separately according to sub‐items, and the total score is calculated. Options A–D are scored as 1–4 points respectively; for items 6 and 7, “no source” is scored as 0 points, and for those with sources, the number of sources is scored. The higher the score of the 10 items, the higher the social support. A total score ≤ 22 indicates a low level, 23–44 indicates a medium level, and 45–66 indicates a high level. In this study, the Cronbach's α coefficient was 0.744, indicating good reliability. The KMO value was 0.751, suggesting that the data were suitable for factor analysis.

#### 3.2.5 The Distress Disclosure Index (DDI)

This scale was developed by Kahn et al. (Kahn and Hessling 2001) in 2001 to measure the degree to which individuals disclose private information such as their troubles to others. Chinese scholar Li Xinmin et al. (Li 2011) translated and revised it into a Chinese version. The scale has a total of 12 items, is single‐dimensional, and uses a Likert 5‐point scoring method. Items 1, 3, 6, 7, 11, and 12 are scored positively, ranging from “strongly disagree” to “strongly agree”, scored as 1–5 points respectively; the remaining items are scored reversely. The total score ranges from 12 to 60 points, with 12–29 points indicating a low level, 30–44 points indicating a medium level, and 45–60 points indicating a high level. In this study, the Cronbach's α coefficient was 0.897, indicating good reliability. The KMO value was 0.927, suggesting that the data were suitable for factor analysis.

### 3.3 Statistical Methods

All statistical analyses were performed using SPSS 25.0. Normally distributed measurement data were described using mean ± standard deviation, non‐normally distributed measurement data were described using median and quartiles, and count data were described using frequency and percentage. Pearson correlation analysis was used to test the pairwise correlations between variables. Mplus 8.3 statistical software was used to conduct mediating effect analysis through the Bootstrap method, and *p* < 0.05 was considered statistically significant. R language was used for sensitivity power analysis to justify the adequacy of the sample size for detecting the hypothesized indirect effects.

## 4 Results

### 4.1 General Information of Postoperative Colorectal Cancer Patients

A total of 165 valid patients were collected in this study, including 92 male patients, accounting for 55.75% of the total; the majority of participants (*n* = 114, 69.09%) were aged over 60 years, and most participants (*n* = 148, 89.70%) were married. Detailed characteristics are presented in (Table 1).

**TABLE 1:** General data of patients after colorectal cancer surgery (*n* = 165).

Variable	Type	Number (*n*)	Proportion
Gender	Male	92	55.75%
	Female	73	44.25%
Age (years)	18–60	51	30.91%
	61–80	114	69.09%
Education	Primary school and below	42	25.46%
	Junior high school	60	36.36%
	High school or technical secondary school	38	23.03%
	Junior college	15	9.09%
	University and above	10	6.06%
Marital status	Married	148	89.70%
	Other situations	17	10.03%
Monthly family income (yuan)	≤ 2000	24	14.55%
	2001–4000	32	19.39%
	4001–6000	44	26.67%
	6001–8000	38	23.03%
	> 8000	27	16.36%
Payment method	Self‐payment	12	15.15%
	Urban medical insurance	58	35.15%
	Rural cooperative medical system	45	19.40%
	Others	50	30.30%
Tumour stage	I	19	11.52%
	II	38	23.03%
	III	98	59.39%
	IV	10	6.06%
Disease type	Colon cancer	70	42.42%
	Rectal cancer	83	50.30%
	Borderline cancer	12	7.28%
Stoma	Yes	28	16.97%
	No	137	83.03%

### 4.2 Scores of Benefit Finding, Self‐Perceived Burden, Self‐Disclosure, and Social Support

The total score of benefit finding in postoperative colorectal cancer patients was (69.73 ± 11.176) points, the total score of self‐perceived burden was (29.74 ± 7.729) points, the total score of self‐disclosure was (35.20 ± 8.772) points, and the total score of social support was (41.25 ± 6.940) points. The scores of each dimension and total scale are shown in (Table 2).

**TABLE 2:** Scores of benefit finding, self‐perceived burden, self‐disclosure and social support (*n* = 165).

Item	Minimum	Maximum	Number of items	Score (points, x ± s)	Average score of items (points, x ± s)
Family Relationship Dimension	4	10	2	6.65 ± 1.198	3.33 ± 0.599
Health Behaviour Dimension	6	14	3	9.81 ± 1.752	3.27 ± 0.584
Personal Growth Dimension	14	33	7	22.58 ± 3.743	3.23 ± 0.535
Acceptance Dimension	6	14	3	9.60 ± 1.742	3.20 ± 0.581
World View Dimension	8	19	4	12.27 ± 2.330	3.07 ± 0.583
Social Relationship Dimension	5	13	3	8.81 ± 1.748	2.94 ± 0.583
Total Score of Benefit Finding	46	100	22	69.73 ± 11.176	3.17 ± 0.508
Total Score of Self‐Perceived Burden	14	46	10	29.74 ± 7.729	2.97 ± 0.773
Total Score of Self‐Disclosure	16	55	12	35.20 ± 8.772	2.93 ± 0.731
Objective Support Dimension	4	16	3	10.03 ± 3.027	3.34 ± 1.009
Subjective Support Dimension	11	30	4	23.07 ± 3.890	5.76 ± 0.972
Utilization Degree of Support Dimension	4	12	3	8.15 ± 2.349	2.71 ± 0.783
Total Score of Social Support	19	57	10	41.25 ± 6.940	4.13 ± 0.694

### 4.3 Correlation Analysis

The results of the correlation analysis of variables showed that benefit finding was negatively correlated with self‐perceived burden and positively correlated with self‐disclosure and social support; self‐perceived burden was negatively correlated with self‐disclosure and social support; self‐disclosure was positively correlated with social support. The scores of each dimension and total scale are shown in (Table 3).

**TABLE 3:** Correlation analysis of benefit finding, self‐perceived burden, self‐disclosure and social support (*n* = 165).

Variables		Benefit finding	Self‐perceived burden	Self‐disclosure	Social support
Benefit Finding	r	1	−0.687	0.712	0.696
	*P*		< 0.001	< 0.001	< 0.001
Self‐Perceived Burden	r	−0.687	1	−0.682	−0.520
	*P*	< 0.001		< 0.001	< 0.001
Self‐Disclosure	r	0.712	−0.682	1	0.585
	*P*	< 0.001	< 0.001		< 0.001
Social Support	r	0.696	−0.520	0.585	1
	*P*	< 0.001	< 0.001	< 0.001	

### 4.4 Mediating Analysis

**FIGURE 2:**
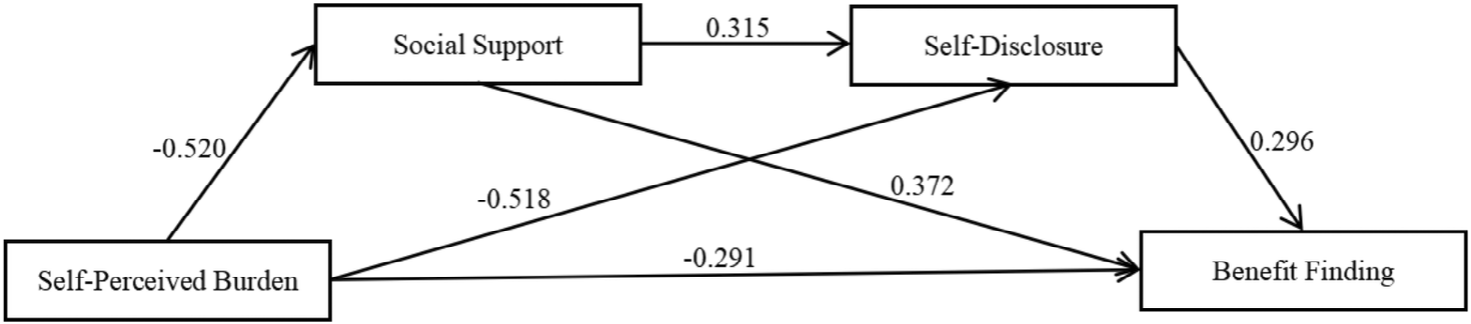
Diagram of the mediating effect of self‐disclosure and social support on the relationship between self‐perceived burden and benefit finding.

The variables were standardized, and Mplus 8.3 statistical software was used to repeat sampling 5000 times through the Bootstrap method to test the predictive relationships and mediating effects among social support, self‐disclosure, self‐perceived burden, and benefit finding. According to the results, it was found that this model was a saturated model, and no requirements were placed on the fit index, only focusing on its path coefficients (Zhang et al. 2019). The results showed that the predictive relationships between the variables were established (Table 4); at the same time, the three paths were tested, and the 95% confidence intervals of all paths did not include 0, indicating that the mediating effects were significant. Among them, the direct effect value was −0.291, accounting for 42.4% of the total effect value; the total mediating effect value was −0.395, accounting for 57.6% of the total effect value. The ratios of the effects of the three mediating paths to the total effect were 22.3%, 28.2%, and 7.1% respectively. The scores of each dimension and total scale are shown in Table 5. The mediating effect model diagram is shown in (Figure 2).

**TABLE 4:** Mediating model tests of self‐perceived burden on benefit finding.

Path	Standardized coefficient	Standard error	*t*	*P*
Self‐Perceived Burden → Benefit Finding	−0.291	0.067	−4.34	< 0.001
Social Support → Benefit Finding	0.372	0.061	6.124	< 0.001
Self‐Disclosure → Benefit Finding	0.296	0.073	4.078	< 0.001
Self‐Perceived Burden → Self‐Disclosure	−0.518	0.067	−7.744	< 0.001
Social Support → Self‐Disclosure	0.315	0.059	5.363	< 0.001
Self‐Perceived Burden → Social Support	−0.52	0.067	−7.806	< 0.001

**TABLE 5:** Mediating effects of social support and self‐disclosure between self‐perceived burden and benefit finding.

Path	Standardized coefficient	Standard error	*t*	Effect percentage	*P*	Lower 2.5%	Upper 2.5%
Social Support → Self‐Disclosure → Benefit Finding	0.093	0.031	3.024	—	0.002	0.044	0.164
Mediating Effect 1: Self‐Perceived Burden → Self‐Disclosure → Benefit Finding	−0.153	0.045	−3.414	22.3%	0.001	−0.252	−0.074
Mediating Effect 2: Self‐Perceived Burden → Social Support → Benefit Finding	−0.193	0.043	−4.54	28.2%	< 0.001	−0.288	−0.121
Mediating Effect 3: Self‐Perceived Burden → Social Support → Self‐Disclosure → Benefit Finding	−0.049	0.017	−2.847	7.1%	0.004	−0.092	−0.023
Direct Effect: Self‐Perceived Burden → Benefit Finding	−0.291	0.067	−4.34	42.4%	< 0.001	−0.423	−0.159
Total Effect	−0.686	0.054	−12.663	100%	< 0.001	−0.791	−0.577

## 5 Discussion

This study employed a cross‐sectional design, with all variables measured at a single time point. Therefore, the mediation analyses should be interpreted as reflecting statistical associations rather than temporal or causal relationships. The ordering of variables in the proposed mediation model was guided by Stress and Coping Theory and previous empirical evidence; however, the cross‐sectional nature of the data does not allow confirmation of temporal sequencing among variables. Future longitudinal or prospective studies are needed to further examine these relationships. Nevertheless, the present findings contribute to understanding the psychosocial factors associated with benefit finding among postoperative colorectal cancer patients. In this section, we discuss the key findings in relation to the existing literature, including the current status of benefit finding, the mediation pathways, and implications for clinical practice.

### 5.1 Status of Benefit Finding in Postoperative Colorectal Cancer Patients

In this study, the total score of benefit finding among postoperative colorectal cancer patients was 69.73 ± 11.176, which was higher than that reported in a previous study focusing on colorectal cancer patients with permanent colostomy (Wang, Chou, et al. 2022). This discrepancy may be attributed to differences in study populations. Patients with stoma have a change in physical integrity, and using a stoma bag for excretion brings a sense of shame to patients, seriously affecting their self‐esteem. It may also be due to the large proportion of elderly patients in this study. Compared with young people, they have more life experiences and can handle stressors more skillfully and face them more calmly, thus perceiving more benefits (Bai et al. 2022). Among all dimensions of benefit finding, the social relationship dimension obtained the lowest average score (2.94 ± 0.583). A possible explanation is that it may be because patients have just undergone surgery, lack the energy to maintain social relationships, and are unwilling to inform their friends about having cancer to avoid causing worry. Future researchers can conduct intervention studies on postoperative colorectal cancer patients targeting the social relationship dimension. By regularly organizing patient exchange meetings, patients can make more friends in the hospital, exchange experiences and feelings, relieve the negative feelings of being unwilling to share with their families, and thus improve the overall benefit finding.

### 5.2 Status of Self‐Disclosure, Self‐Perceived Burden, and Social Support in Postoperative Colorectal Cancer Patients

This study showed that the score of self‐disclosure in postoperative colorectal cancer patients was (35.20 ± 8.772) points, at a medium level of self‐disclosure; the score of self‐perceived burden was (29.74 ± 7.729) points, at a mild level of self‐perceived burden; and the score of social support was (41.25 ± 6.940) points, at a medium level. The score of self‐disclosure was lower than that of gynaecological cancer patients (Mao et al. 2023), which may be related to the different gender compositions of the survey objects. In this study, male patients accounted for 55.75%, and Chinese men are more inclined to reserved and restrained expressions or bear the difficulties and concerns brought by the disease alone without excessive self‐disclosure to others (Dai and Yang 2021). The level of self‐perceived burden in our sample was lower than that in patients with intestinal stoma (Han et al. 2024). Stoma patients require long‐term stoma care and often rely on family assistance, which reduces their self‐worth and aggravates perceived burden. They want to be grateful for the help from others but cannot repay it, resulting in a further increase in self‐perceived burden (Han et al. 2024). At the same time, most participants in this study were elderly patients with relatively narrow social networks; thus, family members became their primary source of social support. Among them, the average score of the subjective support dimension was the highest, at (5.76 ± 0.972) points. Subjective social support mainly reflects an individual's feelings and satisfaction with the support from family, friends, and other social sources and can relieve patients negative emotions. Studies have confirmed that perceived social support plays a more critical role in mental health than objective support, prompting patients to adjust their postoperative mentality and increase their confidence in the future (Lakey and Orehek 2011). Therefore, medical staff should strengthen continuous care for postoperative colorectal cancer patients, improve their self‐care ability, and closely monitor their psychological changes to deliver stage‐based psychological interventions (Liu et al. 2022). It is also important to encourage patients to actively utilize social resources, and organize health education seminars and peer exchange activities to expand their social support system. Meanwhile, family members should be guided to provide adequate companionship and emotional support for patients outside the hospital.

### 5.3 Mediating Effects of Self‐Disclosure and Social Support in the Relationship Between Self‐Perceived Burden and Benefit Finding

#### 5.3.1 Self‐Perceived Burden Was Negatively Associated With Benefit Finding

The findings indicated that self‐perceived burden was negatively associated with benefit finding among postoperative colorectal cancer patients (*r* = −0.687, *p* < 0.001), with the direct effect accounting for 42.4% of the total effect. The present findings suggest a significant negative association between self‐perceived burden and benefit finding. Patients reporting higher levels of self‐perceived burden also tended to report lower levels of benefit finding. Previous studies have reported that lower levels of self‐perceived burden were associated with greater confidence, more positive coping, and higher levels of post‐traumatic growth (Zhang et al. 2022). Post‐traumatic growth and benefit finding both belong to the category of positive psychology. In this study, due to the needs of surgical treatment and postoperative rehabilitation, patients not only have economic burdens but also burdens and pressures regarding subsequent treatment and family care. Patients experiencing greater self‐perceived burden may have fewer psychological resources available for identifying positive changes related to their illness experience, which could help explain the observed association.

#### 5.3.2 Social Support as an Indirect Association Between Self‐Perceived Burden and Benefit Finding

The mediation analysis suggested an indirect association between self‐perceived burden and benefit finding through social support (β = −0.193, *p* < 0.01), accounting for 28.2% of the total effect. These findings indicate that self‐perceived burden is not only directly associated with benefit finding among postoperative colorectal cancer patients but also indirectly affects it through social support. Previous studies have suggested that individuals with greater self‐perceived burden may be less likely to seek or perceive social support (Kong et al. 2024). According to the stress and coping theory (Taylor 1983), social support is a coping resource. According to Stress and Coping Theory, social support is considered an important coping resource that may help individuals manage stress and facilitate psychological adaptation. The present findings suggest that lower self‐perceived burden, greater social support, and higher benefit finding were associated with one another. Social support may represent an important psychosocial factor related to benefit finding among postoperative colorectal cancer patients.

#### 5.3.3 The Role of Self‐Disclosure in the Association Between Self‐Perceived Burden and Benefit Finding

The mediation analysis suggested an indirect association between self‐perceived burden and benefit finding through self‐disclosure (β = −0.153, *p* < 0.01), and this mediating effect accounts for 22.3% of the total effect. These findings suggest that self‐perceived burden was indirectly associated with benefit finding through self‐disclosure among postoperative colorectal cancer patients. Compared with patients with heavier burdens, those with lighter burdens are more willing to actively vent negative emotions and express their own feelings due to less pressure. Self‐disclosure has been described as a coping strategy that may facilitate emotional expression and psychological adjustment (Yao et al. 2024). These findings are broadly consistent with Lazarus and Folkman's Stress and Coping Theory (Taylor 1983). According to Stress and Coping Theory, individuals may use coping resources such as self‐disclosure when responding to illness‐related stress. In the present study, self‐disclosure was statistically associated with both self‐perceived burden and benefit finding. However, the cross‐sectional design does not permit conclusions regarding temporal ordering or causal relationships.

#### 5.3.4 The Joint Role of Social Support and Self‐Disclosure in the Association Between Self‐Perceived Burden and Benefit Finding

The mediation analysis suggested indirect associations involving self‐disclosure and social support in the relationship between self‐perceived burden and benefit finding. The findings suggest that self‐perceived burden, social support, self‐disclosure, and benefit finding were statistically associated in a sequential mediation model (β = −0.049, *p* < 0.01), and this mediating effect accounts for 7.1% of the total effect. In other words, social support and self‐disclosure were each associated with the relationship between self‐perceived burden and benefit finding and were also involved in the sequential mediation model. Previous studies have suggested that lower levels of self‐perceived burden may be associated with better psychological adjustment. Individuals reporting lower self‐perceived burden may perceive greater availability of support from family and friends (Han et al. 2024). At the same time, social support and self‐disclosure are closely related psychosocial resources. Patients who perceive higher levels of social support may be more likely to communicate their experiences and concerns with others, and both factors may be associated with higher levels of benefit finding (Yuan et al. 2023). It should be emphasized that this is a cross‐sectional study. The observed serial associations only represent statistical correlations, rather than definite causal sequences. Longitudinal studies are required in the future to further verify the causal pathways.

## 6 Conclusion

Guided by the stress and coping theory, this study explored the influencing mechanisms of benefit finding among postoperative colorectal cancer patients. Four pathways linking self‐perceived burden to benefit finding were identified. The findings verify that reducing self‐perceived burden is an effective approach to improve benefit finding and provide new insights for subsequent research on positive psychological adaptation among cancer patients. In clinical practice, nurses should regularly assess patients psychological status to prevent excessive self‐perceived burden. Nursing interventions should focus on helping patients build a stable social support system with more companionship from family members. Clinical staff should also create a supportive environment to encourage patients to express inner emotions and release negative feelings. Combined interventions targeting self‐perceived burden, social support, and self‐disclosure can ultimately enhance benefit finding among postoperative colorectal cancer patients.

## 7 Limitations

The sample size of this study (*n* = 165) is relatively sufficient, but it comes from only one hospital, which may limit the accuracy of effect estimation and the universality of results. Although the sensitivity power analysis confirmed that there was *sufficient power* (> 0.90) for the observed medium to large indirect effects, more multicenter studies are needed to confirm the conclusion in the future.

The proportion of non‐ostomy patients in this study was 83.03%, while ostomy patients accounted for less than 17%. Given that ostomy patients are widely considered to have higher postoperative self‐perceived burden (Han et al. 2024) and may differ qualitatively in key psychosocial variables, the chain mediation model identified in this study applies strictly to non‐ostomy patients and should not be directly generalized to the ostomy subgroup. Future research could deliberately increase the proportion of ostomy patients to 40%–50% of the total sample to independently test whether the model holds or examine ostomy status as a potential moderator of the proposed pathways.

The study did not consider that potential confounding factors (such as age, operation time, cancer stage, socio‐economic status) may affect the results of the study, which may lead to bias in the results. Relevant covariates should be included in the analysis of future studies.

In addition, all variables were assessed via self‐report questionnaires at a single time point, which raises the risk of common method bias. To assess this, we conducted Harman's single‐factor test using unrotated exploratory factor analysis. The results revealed 14 factors with eigenvalues > 1, and the first factor accounted for 29.287% of the total variance, which is below the recommended threshold of 40%. This suggests that common method bias did not pose a serious threat to the validity of the findings. Nevertheless, future studies could adopt multi‐method or multi‐informant designs to further minimize this bias.

## 8 Implications for Nursing Practice

First, assessment of self‐perceived burden may be incorporated into routine postoperative nursing evaluations to facilitate early identification of patients at risk. Second, interventions aimed at enhancing social support and encouraging self‐disclosure—such as dual coping interventions—could be considered to support patients psychosocial adjustment and are associated with higher levels of benefit finding (Zhou et al. 2024). Third, the associations observed among self‐perceived burden, social support, self‐disclosure, and benefit finding provide a clinically relevant framework for nurses to monitor patients psychological adaptation.

## Author Contributions

YiLong ZHOU and HuiFang GUO conceived and designed the study. JingJing GUO and Jia WEN performed data collection and statistical analysis. YiLong ZHOU drafted the manuscript. YaJing WANG critically revised the manuscript for important intellectual content. All authors read and approved the final manuscript. YiLong ZHOU and YaJing WANG contributed equally to this work and are therefore co‐first authors.

## Funding

This research received no specific grant from any funding agency in the public, commercial, or not‐for‐profit sectors.

## Ethics Statement

This study was approved by the ethics committee of the First Affiliated Hospital of Dalian Medical University on January 30, 2024 (PJ‐KS‐KY‐2024‐74). Written informed consent was obtained from all participants.

## Conflicts of Interest

The authors declare no conflicts of interest.

## Data Availability

The data that support the findings of this study are available from the corresponding author upon reasonable request.
